# In this month’s Bulletin

**DOI:** 10.2471/BLT.22.000522

**Published:** 2022-05-01

In editorials this month, Stefan Listl et al. (294) call for harnessing citizens’ values through deliberative processes to improve oral health systems. Samira Choudhury et al. (295) discuss how lessons from the COVID-19 pandemic create opportunities to manage antimicrobial resistance.

Lynn Eaton (298–299) reports on antimicrobial misuse during the COVID-19 pandemic and the lack of adherence to treatment protocols. Parameswaran Iyer talks to Gary Humphreys (300–301) about how to transform water, sanitation and hygiene practices through behavioural change interventions.

## Malawi

### Improving child survival 

King et al. (302–314) identify prognostic associations with hypoxaemia or hypoglycaemia.

## China

### Testing helmet campaigns 

Ning et al. (329–336) measure helmet use by cyclists and motorcyclists. 

## Sub-Saharan Africa

### Financial hardship and health care

Eze et al. (337–351) estimate incidence and trends of catastrophic expenditure.

## Global

### Too many or too few caesarean sections?

Opiyo et al. (352–354) present a global data-sharing platform for facility-level rates. 

### Ensuring vaccine equity

Yoo et al. (315–328) track COVID-19 vaccine allocation and distribution through COVAX. 

